# Overweight increases perioperative spinal surgery complications: a single-center retrospective study

**DOI:** 10.1186/s12891-023-06217-z

**Published:** 2023-02-06

**Authors:** Masahiro Hirahata, Youichi Yasui, Muneyoshi Fujita, Keisuke Ishii, Hirotaka Kawano, Tomoaki Kitagawa

**Affiliations:** 1 Department of Orthopaedic Surgery, Teikyo University School of Medicine, 2-11-1 Kaga, Tokyo, Itabashi-Ku 1738605 Japan; 2 Trauma and Reconstruction Center, Teikyo University Hospital, Tokyo, Japan

**Keywords:** Obesity, Overweight, Spinal surgery, Complication

## Abstract

**Background:**

The World Health Organization (WHO) defines a person with a body mass index (BMI) greater than or equal to 25 kg/m^2^ as overweight. Being overweight is a lifestyle-related disease; however, little is known about the impact of overweight on the perioperative complications of orthopedic surgery. This study aimed to define the effect of overweight on the perioperative complications of spinal surgery.

**Methods:**

This retrospective case series study reviewed 269 consecutive patients who underwent spinal surgery. These patients were divided into the overweight (OW) and non-overweight (NOW) groups. Age, BMI, surgical time, blood loss, and perioperative complications were evaluated and compared between the groups.

**Results:**

There were 117 patients (43%) in the OW group and 152 (57%) in the NOW group. Cervical surgery was performed in 72 cases, thoracic surgery in 34, and lumbosacral surgery in 159. The surgical time was significantly longer in the OW group than in the NOW group (204.6 ± 98 min vs. 175 ± 75 min; *p* = 0.01). Blood loss was greater in the OW group than in the NOW group (446.8 ± 447.9 mL vs. 279 ± 296.5 mL; *p* = 0.00). Durotomy was more frequent in the OW group than in the NOW group (10 vs. 3 cases; *p* = 0.02). There was no difference in complications other than durotomy.

**Conclusions:**

OW patients had longer surgical time, more blood loss, and more frequent durotomy than NOW patients. These findings indicate that overweight increases perioperative complications of spinal surgery.

## Background

Spinal surgery is known to be one of the most affected surgeries by obesity. Patients with obesity undergoing spinal surgery have a high risk of postoperative complications, particularly surgical site infection and venous thromboembolism [1,2]. According to the hypothesis of those studies, patients with obesity, defined as having a BMI ≧ 30 kg/m^2^, have a high risk of surgical complications. On the other hand, little is known about the adverse effects of overweight (OW) on surgery-related perioperative complications.

In 1995, the World Health Organization (WHO) suggested four obesity categories based on the body mass index (BMI): underweight, < 18.5 kg/m^2^; normal, 18.5– < 25 kg/m^2^; overweight (OW), 25– < 30 kg/m^2^; and obese, > 30 kg/m^2^ [3]. The number of individuals with obesity has rapidly increased. Furthermore, the worldwide prevalence of obesity has nearly tripled between 1975 and 2016 [3]. The WHO suggested that OW and obese are significantly related to lifestyle-related diseases, such as hypertension and diabetes mellitus [4,5], which have also been associated with an increase in the incidence of cancer [6]. Musculocutaneous disorders, such as osteoarthritis, affect load-bearing joints and cause spinal deformity. In addition to the fact that joints are broken by the excessive load, muscle, tendon, and joint problems caused by the release of various inflammatory mediators by mast cells have become evident in recent years [7].

Numerous studies have demonstrated the impact of obese on perioperative complications. The incidence of perioperative complications in thoracoabdominal surgery has been well documented [8,9]. In orthopedic surgeries, patients with obese had significantly lower patient-reported outcome measures than non-obese patients following hip arthroscopic surgery. Furthermore, the total hip arthroplasty conversion and complication rates were 2.4 and 3.2 times higher in patients with obese than non-obese patients, respectively [10]. A correlation between increased BMI and perioperative outcomes has been reported, particularly surgical site infections and renal and respiratory complications, in patients undergoing total knee arthroplasty [11]. The risk of total ankle arthroplasty failure has increased in patients with obese [12]. Midgley et al. highlighted in a literature review that this growing cohort of struggling patients is currently supported by existing care frameworks. However, they concluded that more research in this subject is warranted [13]. However, these studies also had not examined the effects of OW.

This respective study aimed to determine the effect of OW on perioperative complications of spinal surgery. We hypothesized that the WHO standard of OW adversely affects and increases perioperative spinal surgery complications.

## Methods

### Study subjects

This study was approved by Teikyo University Ethical Review Board for Medical and Health Research Involving Human Subjects (19–174) and conducted according to the World Medical Association Declaration of Helsinki. All individuals included in this study provided written informed consent.

A retrospective chart review of 269 consecutive patients who underwent spinal surgery in the author’s institution between October 2016 and September 2018 was conducted. All surgeries that involved pre- and postoperative care were performed by one spine surgeon with more than 20 years of experience.

The inclusion criteria were patients who underwent elective surgeries with more than 1 year of follow-up after surgery. Surgical sites included the cervical, thoracic, and lumbosacral spine. The exclusion criteria were patients who underwent revision and secondary surgeries for debridement or repair of the dural membrane following primary surgeries. Patients who underwent anterior approach surgeries were excluded.

### Evaluation

All patients were classified into the OW (BMI ≥ 25.0 kg/m^2^) and non-overweight (NOW) (BMI < 25.0 kg/m^2^) groups according to the WHO definition. Patients were also subgrouped into those who underwent cervical, thoracic, or lumbosacral surgery and those who underwent instrumentation and noninstrumentation surgeries. Age, BMI, surgical time, blood loss, and perioperative complications were obtained, and these data were compared between the groups. The receiver operating characteristic (ROC) curves of the sensitivity and specificity of BMI for all perioperative complications and the most common complication were plotted.

### Statistical analysis

Categorical variables such as sex and complications were compared using the chi-squared test (or Fisher’s exact test, where appropriate). The threshold for statistical significance was a *P*-value < 0.05. Student’s t-test was employed for age, BMI, surgical time, and blood loss based on OW or NOW. In this case, statistical significance was also a *P*-value < 0.05. ROC curves were used to elucidate the cut-off values of BMI in complications, especially durotomy. Statistical analyses were conducted using the SAS 9.4 software for Windows (SAS Institute Inc., Cary, NC, USA).

## Results

### *Patient demographics *(Table 1*)*

**Table 1:** Analyzed variables with percentages

	All (*n* = 269)	Percentage
Age	64 (7 - 90)	
Female sex	90	33.5
BMI	24.2 (16.2 - 37.5)	
OW	117	43.5
Level		
Cervical	72	26.8
Thoracic	38	14.1
Lumbosacral	159	59.1
Instrumentation	148	55.0
Surgical time	188 (35 - 721)	
Blood loss	352 (10 - 2400)	
Complication	46	17.1
Durotomy	13	4.8
Comorbidity		
Hemodialysis	14	5.2
Rheumatoid arthritis	5	1.9
Bronchial asthma	2	0.7

This study included 90 women and 179 men, with a mean age of 63.6 years (range, 7–90) and a mean BMI of 24.2 kg/m^2^ (range, 16.2–37.5). Only one patient was morbidly obese who has a BMI of 40 or higher or a BMI of 35 or higher and is experiencing obesity-related health conditions.

### *Outcomes in the OW and NOW groups *(Table 2)

**Table 2:** Overweight

	Overweight		*P* value
	( +); *n* = 117	(-); *n* = 152	
Age	61.8 ± 16.6	65.1 ± 18.0	0.04
Female sex	36	54	0.44
BMI	27.5 ± 2.3	21.6 ± 2.3	0.00
Surgical time	204.6 ± 98.0	175.0 ± 75.0	0.01
Blood loss	446.8 ± 447.9	279 ± 296.5	0.00
Complication	25	21	0.14
Durotomy	10	3	0.02
Cerebrospinal fluid leakage	0	1	0.77
Deep wound infection	4	1	2.76
Epidural hematoma	4	1	2.76
Neurological complications	1	3	0.57
Implant failure	0	1	0.77
Cerebral disease	1	2	0.13
Death	0	1	0.77
others	5	8	0.14

There were 117 patients (43%) in the OW group and 152 (57%) in the NOW group. In the OW group, there were 81 women and 36 men, with a mean age of 61.8 years (range, 7–86) and a mean BMI of 27.5 kg/m^2^ (range, 25.0–37.5). The NOW group had 54 women and 98 men, with a mean age of 65.1 years (range, 17–90) and a mean BMI of 21.6 kg/m^2^ (range, 16.2–24.9).

Surgical time was longer in the OW group than in the NOW group (204.6 ± 98 vs. 175 ± 75 min). There was more blood loss in the OW group than in the NOW group (446.8 ± 447.9 vs. 279 ± 296.5). A total of 46 patients had a perioperative complication, including 13 durotomy cases, five cases of deep wound infections, and five cases of epidural hematoma. Of those, durotomy was more frequent in the OW group than in the NOW group (10 vs. 3).

### BMI cut-off value for complications

**Fig. 1:**
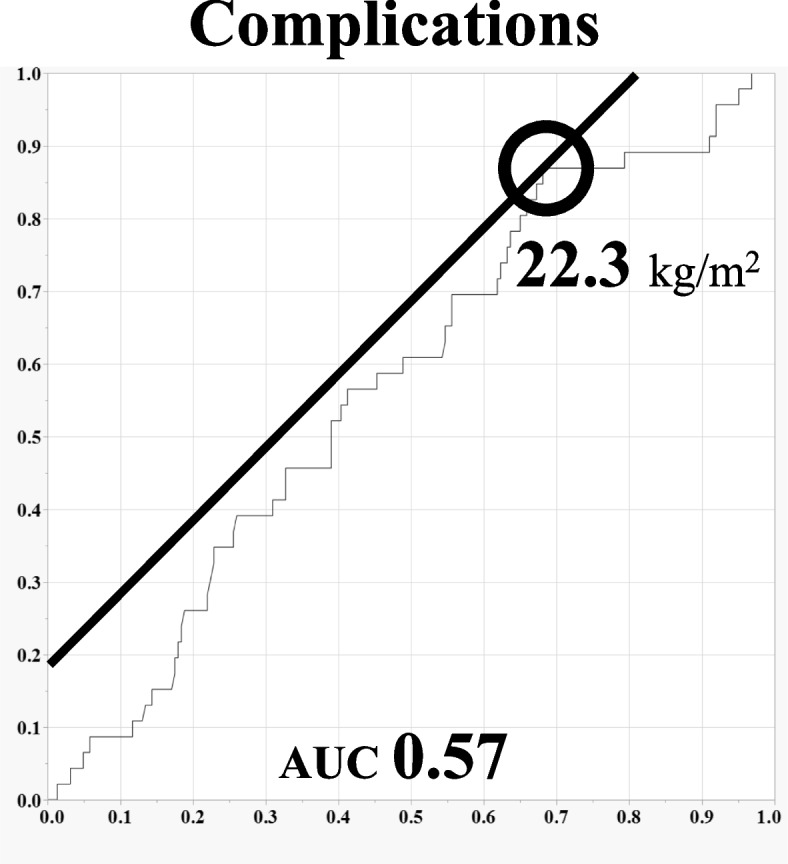
The ROC curve of BMI for all perioperative complications

**Fig. 2:**
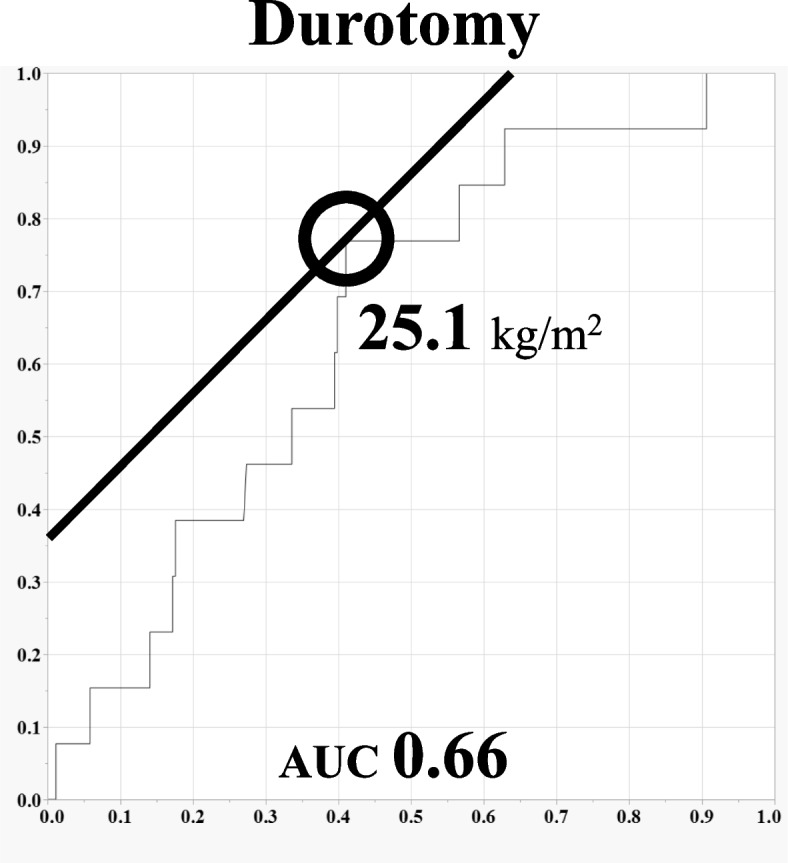
The ROC curve of BMI for durotomy

The ROC curve showed that the BMI cut-off value was 22.3 kg/m^2^ for a perioperative complication and 25.1 kg/m^2^ for durotomy (Figs. 1 and 2).

### Subgroup analysis

**Fig. 3:**
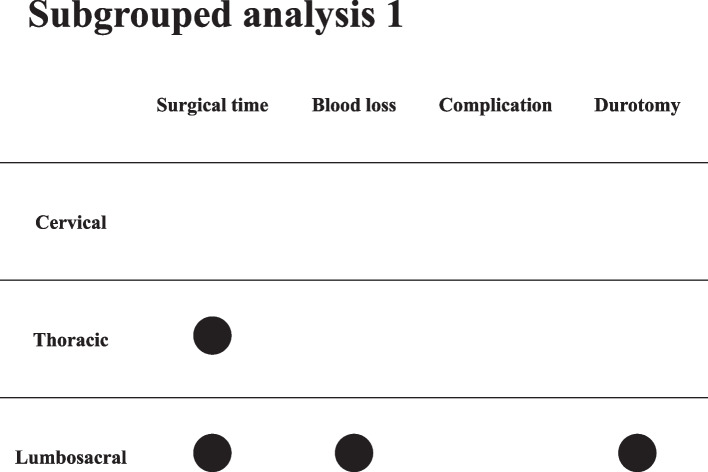
Subgrouped analysis 1: surgical site

**Fig. 4:**
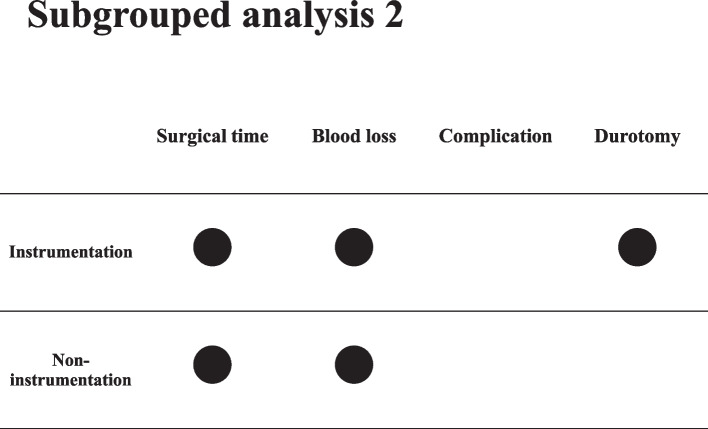
Subgrouped analysis 2: instrumentation

Cervical surgery was performed in 72 cases, thoracic surgery in 34, and lumbosacral surgery in 159. The subgroup analysis of lumbosacral surgery between the OW and NOW groups revealed a significant difference in surgical time, blood loss, and durotomy (Fig. 3). Instrumentation surgery was required in 148 cases and noninstrumentation surgery in 121. The subgroup analysis of only instrumentation surgery between the OW and NOW group revealed a significant difference in surgical time, blood loss, and durotomy (Fig. 4).

## Discussion

The outcomes in this retrospective study based on a single-center cohort demonstrated that surgical time, blood loss, and incidence of durotomy significantly increased in OW patients. The BMI cut-off value for durotomy was also high in OW patients. A consensus on the impact of OW on perioperative complications in elective surgeries has not been established despite the influence of lifestyle-related diseases, such as hypertension and diabetes mellitus [4,5]. The unique feature of this study in comparison with previous ones was the focus on OW. Most previous studies on the effect of increased body size on perioperative compilations focused on the differences between patients with and without obesity in mucocutaneous surgeries [14]. To the best of our knowledge, evidence has been spared on the effect of OW.

The prevalence of OW is rapidly increasing [15], and we believe that the outcomes in our study can serve as a reference for physicians to be attentive to perioperative complications in spinal surgery, not only in cases of obesity but also in OW.

Regarding surgical time and blood loss, we found that OW patients had longer operative times and greater surgical blood loss than NOW patients. A recent systematic review and meta-analysis conducted by Goyal et al. demonstrated that patients with obesity had longer operative times and greater surgical blood loss than non-obese patients in elective lumbar spinal surgery with a diagnosis of degenerative lumbar spine disease [14]. While Goyal’s study focused on patients with obesity, this study focused on both OW and individuals with obesity. Our findings indicate that even in OW patients, surgical time and blood loss may be increased. We consider that the thick subcutaneous adipose tissue affected the operative times and surgical blood loss. Jackson et al. reported that patients with obesity had thick subcutaneous adipose tissue, making it difficult to develop and maintain the operative field. This situation resulted in longer surgical times and greater surgical blood loss [2]. OW patients had longer surgical time and more blood loss than patients with normal weight because the subcutaneous adipose tissue thickens with BMI [16].

This study investigated the incidence of dural tear in elective spinal surgery. A nationwide survey of perioperative complications in all spinal surgeries, which included 31,380 patients, showed that the perioperative complication with the highest incidence was dural tear, which occurred in 2.1% of patients [17]. The incidence rate of dural tear in this study was 4.8%, and OW was independently associated with an increased likelihood of dural tear. This may be because OW patients have thicker subcutaneous tissue than NOW patients. Additionally, the distance from the skin to the dura mater is more significant, making it difficult to achieve an adequate field of vision and intraoperative manipulation. In addition to the outcomes of this study that focuses on OW patients, the influence of obesity on the incidence of dural tear has also been investigated. In Goyal’s systematic review and meta-analysis, the incidence of dural tear was not significantly different between patients with obesity and non-obese patients (MD = 0.83, 95% CI = 0.56–1.23, *P* = 0.48, I^2^ = 64%). On the other hand, the prospective comparative study conducted by Murphy, which was not included in Goyal’s systematic review, suggested that obesity was independently associated with an increased likelihood of a dural tear on adjusted analysis [18]. In Murphy’s study, a dural tear requiring repair occurred in 0.6% of 104,930 patients. Murphy et al. suggested that the increase in dural tear was due to the difficulty developing and maintaining the operative field in patients with obesity and the vulnerability of the dura tear due to various inflammatory mediators from mast cells [7]. Because dural tears were affected by age and other factors, further research on the effect of OW and obesity on dural tears is warranted.

We consider that the adverse effect of OW may be significant in lumber spinal surgery. The subgroup analysis revealed that OW patients who underwent lumbosacral surgery had longer surgical times, more blood loss, and more significant durotomy than NOW patients but not those who underwent cervical and thoracic spinal surgeries. The subcutaneous tissue of the lumbosacral spine is thicker than that of the cervicothoracic spine [2]. Additionally, Kim et al. reported that the abdominal pressure was higher in the lumbosacral spine than in the cervicothoracic spine [19]. These factors may induce adverse events during OW lumber spinal surgery. Among spinal surgeries, lumbar spinal surgery is the most common.

The outcome of the subanalysis indicated that special attention should be paid to OW patients. The subgroup analysis was conducted on both instrumentation and noninstrumentation surgeries. The number of dural tears in OW patients who underwent instrumentation surgery was higher than those who underwent noninstrumentation surgery. In a previous prospective cohort study, Smorgick et al. demonstrated no significant difference in the incidence of dural tear between patients who underwent decompression alone and those who underwent decompression and instrumented spinal fusion [20]. In their study, no conclusions were drawn due to the low number of dural tears. Previous studies that determined dural tear predictors in spinal surgery did not investigate the association between dural tear and obesity [21,22]. The instrumentation process for patients with obesity might be complicated for surgeons due to the deep operative field because of the thick subcutaneous tissue.

Our study suggests a BMI cut-off value of 22.3 kg/m^2^ for perioperative complications and 25.1 kg/m^2^ for durotomy. No previous studies have investigated BMI cut-off values for spinal surgery complications. Compared with a previous study on obesity in spinal surgeries, the patients included in this study were relatively light in weight ^[[[14]]]^. The results are likely to differ from one study population to another but can be helpful information for similar populations.

This study has several limitations. First, a small number of the patients were recruited from a single institution. The elective spinal surgeries were not randomly performed. Because they are elective surgeries, OW and patients with obesity tend not to be selected, thus leading to selection bias. Second, any retrospective design introduces an element of uncertainty. The medical record data may be erroneous or missing, and clinical information may be lacking. The information about surgeries was significantly incomplete, such as fusion, approaches, and pathologies. There is also insufficient information on comorbidities. Third, functional outcomes were not evaluated. Finally, the thickness of the subcutaneous adipose tissue was not measured. Due to the retrospective non-randomized nature of the study, a multicenter, prospective randomized study is required to confirm the effect of OW during spinal surgery.

## Conclusions

Patients with a BMI greater than or equal to 25 kg/m^2^ had longer surgical times, more blood loss, and a significant number of durotomy than those with normal weight. The cut-off value of BMI was 22.3 kg/m^2^ for perioperative complication and 25.1 kg/m^2^ for durotomy. The results of this study can be a valuable reference for physicians to be attentive to perioperative complications of spinal surgery, not only in patients with obesity but also in overweight patients.

## Data Availability

The datasets generated during and analyzed during the current study are not publicly available due to disagreement of participants for their data to be shared but are available from the corresponding author on reasonable request.

## Abbreviations

WHO: World Health Organization
BMI: Body mass index
OW: Overweight
NOW: Non-overweight
ROC: Receiver operating characteristic
